# Scoring pleurisy in slaughtered pigs using convolutional neural networks

**DOI:** 10.1186/s13567-020-00775-z

**Published:** 2020-04-10

**Authors:** Abigail R. Trachtman, Luca Bergamini, Andrea Palazzi, Angelo Porrello, Andrea Capobianco Dondona, Ercole Del Negro, Andrea Paolini, Giorgio Vignola, Simone Calderara, Giuseppe Marruchella

**Affiliations:** 1 Faculty of Veterinary Medicine, University of Teramo, Loc. Piano d’Accio, 64100 Teramo, Italy; 2 AImageLab, University of Modena and Reggio Emilia, Via Vivarelli 10/1, 41125 Modena, Italy; 3 Farm4Trade s.r.l., Via Marino Turchi, 66100 Chieti, Italy

## Abstract

Diseases of the respiratory system are known to negatively impact the profitability of the pig industry, worldwide. Considering the relatively short lifespan of pigs, lesions can be still evident at slaughter, where they can be usefully recorded and scored. Therefore, the slaughterhouse represents a key check-point to assess the health status of pigs, providing unique and valuable feedback to the farm, as well as an important source of data for epidemiological studies. Although relevant, scoring lesions in slaughtered pigs represents a very time-consuming and costly activity, thus making difficult their systematic recording. The present study has been carried out to train a convolutional neural network-based system to automatically score pleurisy in slaughtered pigs. The automation of such a process would be extremely helpful to enable a systematic examination of all slaughtered livestock. Overall, our data indicate that the proposed system is well able to differentiate half carcasses affected with pleurisy from healthy ones, with an overall accuracy of 85.5%. The system was better able to recognize severely affected half carcasses as compared with those showing less severe lesions. The training of convolutional neural networks to identify and score pneumonia, on the one hand, and the achievement of trials in large capacity slaughterhouses, on the other, represent the natural pursuance of the present study. As a result, convolutional neural network-based technologies could provide a fast and cheap tool to systematically record lesions in slaughtered pigs, thus supplying an enormous amount of useful data to all stakeholders in the pig industry.

## Introduction

Diseases of the respiratory system are among the leading causes of economic loss in farm animal breeding, due to increased mortality, decreased daily weight gain and the cost of veterinary care. The impact of respiratory diseases is particularly relevant in growing/finishing pigs raised indoors with high stock density [1]. The aetiology of porcine respiratory diseases is usually multifactorial, an important role being played by infectious agents, which often act together to cause the so-called “porcine respiratory disease complex” (PRDC). The severity and outcomes of PRDC are greatly influenced by the immune status of the animals, by environmental factors impairing the efficacy of the mucociliary barrier of the airways (e.g. the level of ammonia and dust), as well as by a number of managerial factors (e.g. overcrowding, pigs’ flow, biosecurity strategies), which can increase the infectious load and break the balance between herd immunity and the pathogens [2, 3].

*Mycoplasma hyopneumoniae* (*M. hyopneumoniae*) and *Actinobacillus pleuropneumoniae* (*A. pleuropneumoniae*) are among the most important respiratory pathogens in intensively bred pigs, causing enzootic pneumonia (EP) and porcine pleuropneumonia, respectively [4–7]. *Actinobacillus pleuropneumoniae* can cause severe, rapidly fatal fibrinous-hemorrhagic and necrotizing pleuropneumonia in pigs; in survivors, necrotic sequestra and chronic adhesive pleuritis can persist as sequelae [6, 7].

Considering the lifespan of pigs (usually lasting between 5 and 10 months), *M. hyopneumoniae* and *A. pleuropneumoniae* related lesions can still be evident at slaughter, where they can be usefully recorded and scored with very high prevalence values [8, 9]. Therefore, the slaughterhouse represents a key check-point to assess the health status of pigs, in addition to data collected in the herds (e.g. necropsy findings) or resulting from laboratory tests (e.g. serological surveys). The registration of lesions at the abattoir provides unique and valuable feedback to the farm, as well as an important source of data for epidemiological studies [9–11].

Several scoring methods have been developed over the last decades, in order to estimate the impact of diseases in slaughtered pigs, a special emphasis having been placed upon EP-like lesions [12] and pleurisy [13]. As far as pleurisy is concerned, the “*slaughterhouse pleurisy evaluation system*” (SPES) grid is widely used to quantify the impact of *A. pleuropneumoniae* infection. According to the SPES grid, a higher score is given to pleurisy of the diaphragmatic lung lobes [14], which are typically affected in the course of porcine pleuropneumonia [6]. Considering that pleurisy usually affects both pleural sheets, a new scoring method (“*pleurisy evaluation of the parietal pleura*”, PEPP) has been recently developed, based on the inspection of the parietal pleura. Similarly to the SPES grid, the PEPP method also attributes a higher score to the lesions affecting the caudal portion of the chest wall. The SPES and PEPP methods demonstrated to provide well-matching results, the PEPP method being also effectively applicable on digital images [15].

Generally, the ideal scoring method should be simple, fast, easily standardisable, providing suitable data for statistical analysis. Overall, methods currently available to score EP-like lesions and pleurisy at slaughter well fit such requirements. Notwithstanding this, scoring lesions in slaughtered pigs represents a very time-consuming and costly activity, thus making difficult, if not impossible, their systematic recording. Furthermore, abattoir-related and inter-observer variations should also be considered, highlighting the need to standardise all the operative procedures [9, 11].

In this respect, artificial intelligence (AI) based technologies could offer very promising opportunities. Artificial intelligence is a discipline aiming to develop intelligent agents, i.e. machines that can perceive the environment and take action to maximise their success regarding a defined target [16]. Several approaches have been pursued from the beginning of the AI research era to create machines that can simulate human intelligence. At present, the statistical learning approach appears as the dominant methodology, thanks to the success of deep learning (DL), especially in the field of visual object recognition [17]. Deep learning is a subset of machine learning and is based on networks of highly interconnected computer processors (so-called “neurons”), capable of performing parallel computations for data processing and knowledge representation [18].

Over the last few decades, several attempts have been made to apply DL to human health and medicine [19, 20], mostly in the field of diagnostic imaging [21–23]. To the best of our knowledge, AI has never been applied to the identification and quantification of gross lesions in animals. The present study has been carried out to train an AI-based system, aiming to automatically score pleurisy in slaughtered pigs. The automation of lesion scoring would be extremely helpful to enable a systematic examination of all slaughtered livestock, positively influencing herd management, animal welfare and profitability.

## Materials and methods

### Animals

A total of 5902 porcine half-carcasses were included in the present study, between November 2017 and April 2019. Pigs (9–11 months of age; 150–180 kg) were regularly slaughtered in abattoirs located in Central and Northern Italy, under different field conditions (i.e. lighting, background, speed and features of the slaughter chain, etc.).

### Photo acquisition of half-carcasses, pleurisy scoring and data recording

The inner surface of all half-carcasses, including the chest wall, was photographed using different smartphone cameras, under routine field conditions. In particular, pictures were taken along the slaughter line with the half-carcasses hanging upside down, after the removal of viscera and showering. Such pictures were then carefully evaluated by two skilled veterinarians and pleurisy scored through the evaluation of the parietal pleura. In order to obtain a suitable number of pictures per each score, the PEPP method was simplified as follows:

- Absence of pleurisy = 0 points (class I);
- Pleurisy affecting the cranial chest wall, from the 1^st^ to the 5^th^ intercostal space = 1 point (class II);
- Pleurisy affecting the caudal chest wall, from the 6^th^ to the last intercostal space = 2 points (class III);
- Pleurisy affecting both the cranial and caudal chest walls = 3 points (class IV).

**Figure 1:**
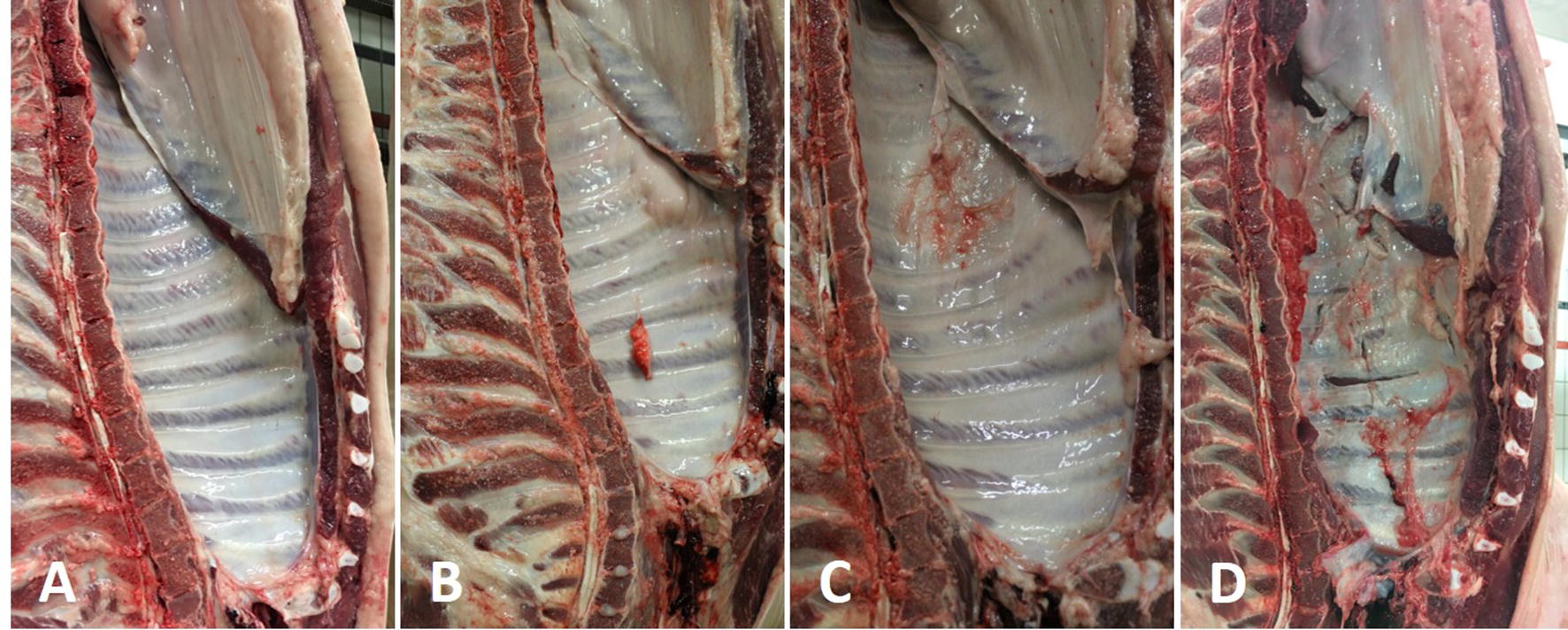
**Scoring pleurisy according to the simplified PEPP method. A** In healthy half-carcass, the parietal pleura appears smooth, wet and transparent, the intercostal spaces being easily appreciated. **B** A small fragment of lung adheres to the parietal pleura, at the level of the 4^th^ intercostal space (score = 1 point). **C** Pleurisy affects the caudal intercostal spaces; at this level, the parietal pleura appears greyish-to-reddish and rough (score = 2 points). The entire parietal pleura is affected by pleurisy, lung fragments adhering to the chest wall (score = 3 points).

Explanatory pictures are shown in Figure 1. All the scores were agreed upon by the two veterinarians and recorded on a Microsoft Excel spreadsheet.

### Photo annotation

**Figure 2:**
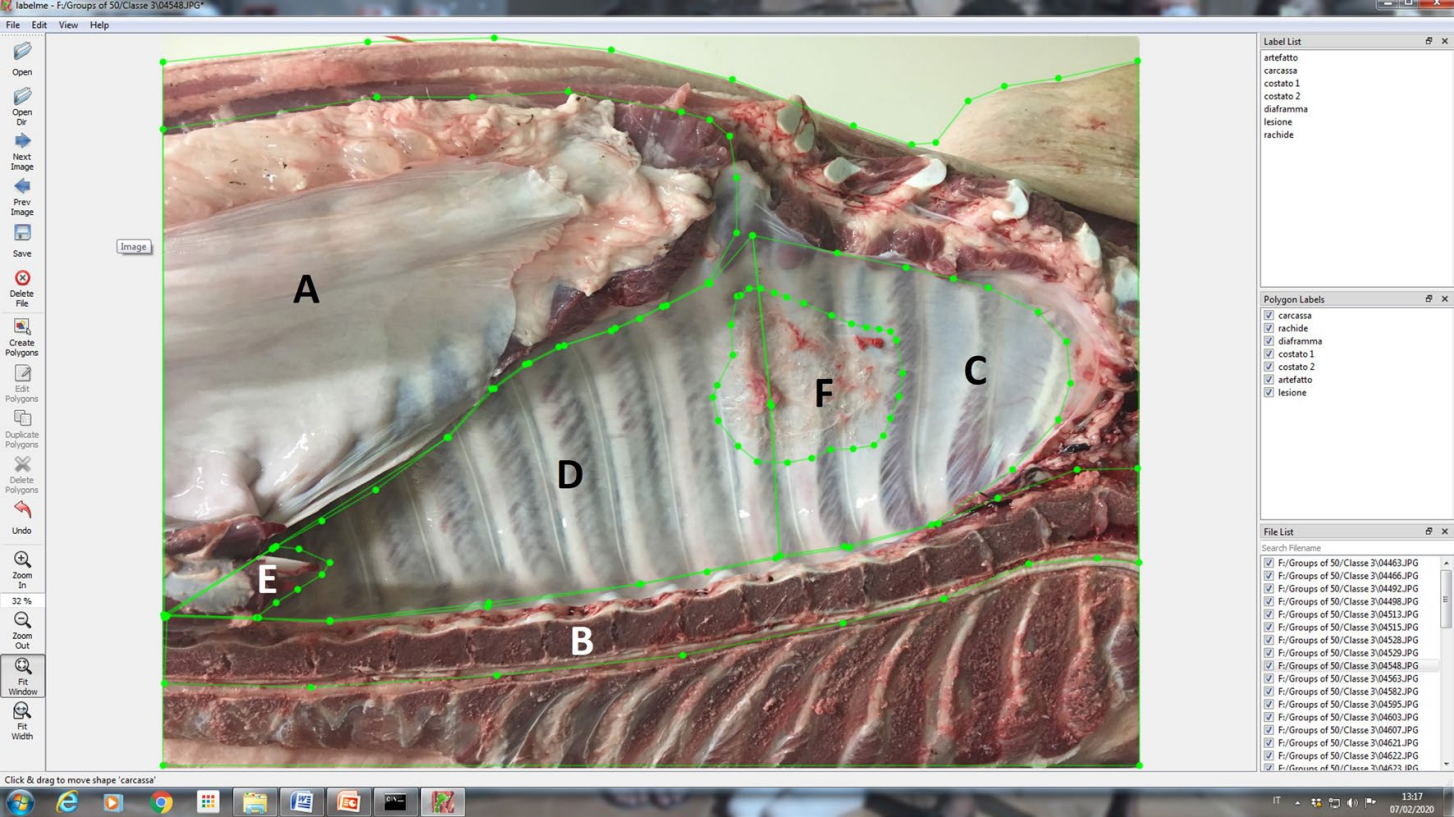
**Segmentation mask using a suitable annotation tool: an example.** In this picture, the following features have been annotated, along with the entire silhouette of the half carcass: diaphragm (**A**), vertebral bodies (**B**), cranial chest wall (**C**), caudal chest wall (**D**), artefact (i.e. a portion of diaphragm covering the chest wall; **E**), pleurisy straddling both areas of the chest wall (**F**).

The same two veterinarians annotated all the above pictures with segmentation masks, using a dedicated open-source image annotation tool [24]. In particular, the following districts were annotated for the task: half-carcass, vertebral bodies, diaphragm, cranial chest wall (from the 1^st^ to the 5^th^ intercostal space), caudal chest wall (from the 6^th^ to the last intercostal space), artefacts (i.e. presence of blood, portions of blood vessels, kidney, liver or fat partially covering the chest wall), pleural lesions (Figure 2).

### DL-based method employed

**Figure 3:**
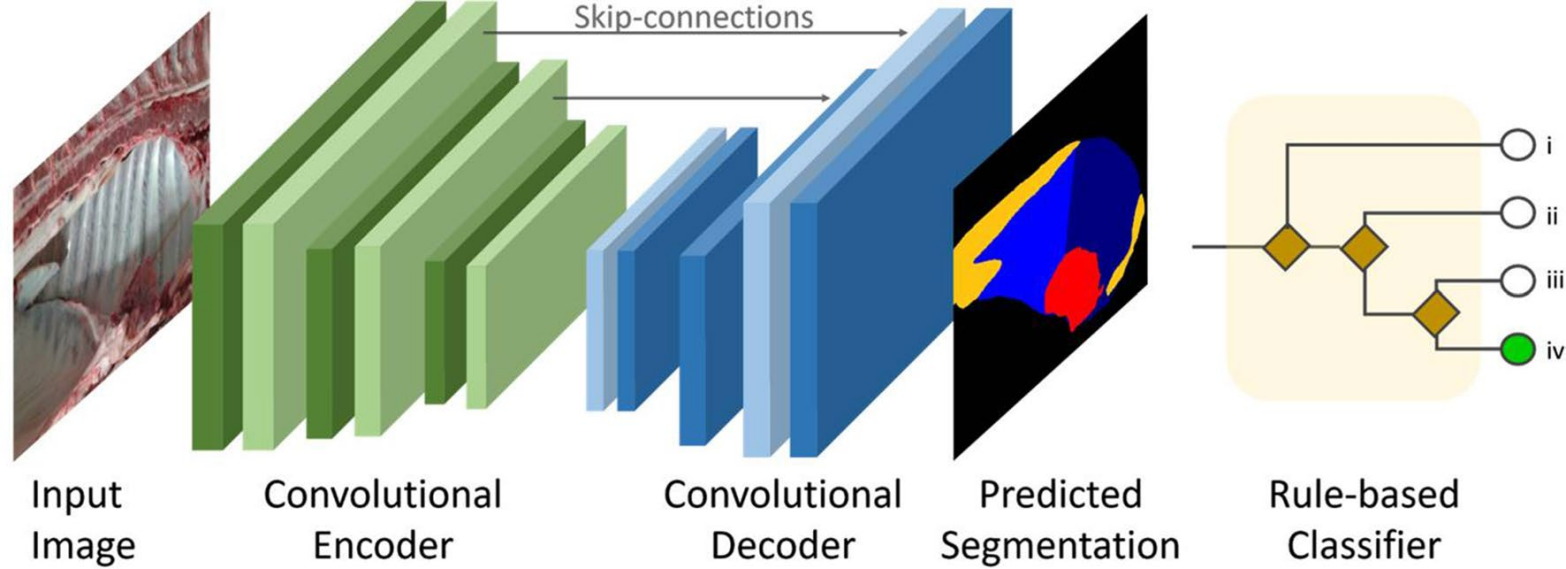
**Overview of the model architecture.** The input image is presented on the left. The CNN has been divided into two components to enhance readability. The convolutional encoder (green) translates the image into a compressed features vector. Then, the convolutional decoder (blue) converts this vector into the final segmentation layers, which can be compared with the ones annotated by the experts. The rule-based classifier is depicted on the right. Given the predicted segmentation layers, this module casts them to one of the 4 classes of interest.

The architecture of the DL method employed herein is graphically shown in Figure 3. It is based on so called “convolutional neural networks” (CNNs) and stems from a network, called “U-Net”, the latter being a convolutional network architecture for fast and precise segmentation of images [25].

Convolutional neural networks represent the state of the art for data driven applications and consist of stacking convolutional layers. More in detail, the present CNN is composed of two sub-modules: namely, an encoder and a decoder.

The encoder has been heavily modified, replacing the U-Net standard with convolutional layers derived from the ResNet-34 model [26]. This allows pre-training on vast classification datasets, permitting to transfer part of that knowledge into our task, by means of simply replacing the encoder layers with ones from a network trained on one such dataset.

The decoder is composed of four convolutional blocks, each one with the same number of channels as the corresponding ResNet-34 layer in the encoder path. Using this approach, we can include paths between the so called “skip-connections”. The name refers to the fact that these are shortcuts between the encoder and the decoder, which promote the flow of information. Intuitively, information such as the general shape can immediately “jump” between the input and output of the CNN, without being processed by every layer.

The network receives as input one RGB (red, green and blue) image and produces segmentation layers as output. Each layer represents one of the anatomical districts of interest and consists of binary values. It is important to refer the final result to the original image, meaning that the first has the same spatial resolution as the latter. If a pixel has a value of 0, then the corresponding anatomical structure is not present in that location. On the contrary, a value of 1 determines the presence of the structure for that location. It is worth mentioning that even if a structure is partially covered by another one in the input image (e.g. a lesion covering one of the chest wall), the network is trained to produce a full structure, instead of one presenting holes. This is necessary for the second stage of our pipeline, i.e. the rule-based classifier.

**Figure 4:**
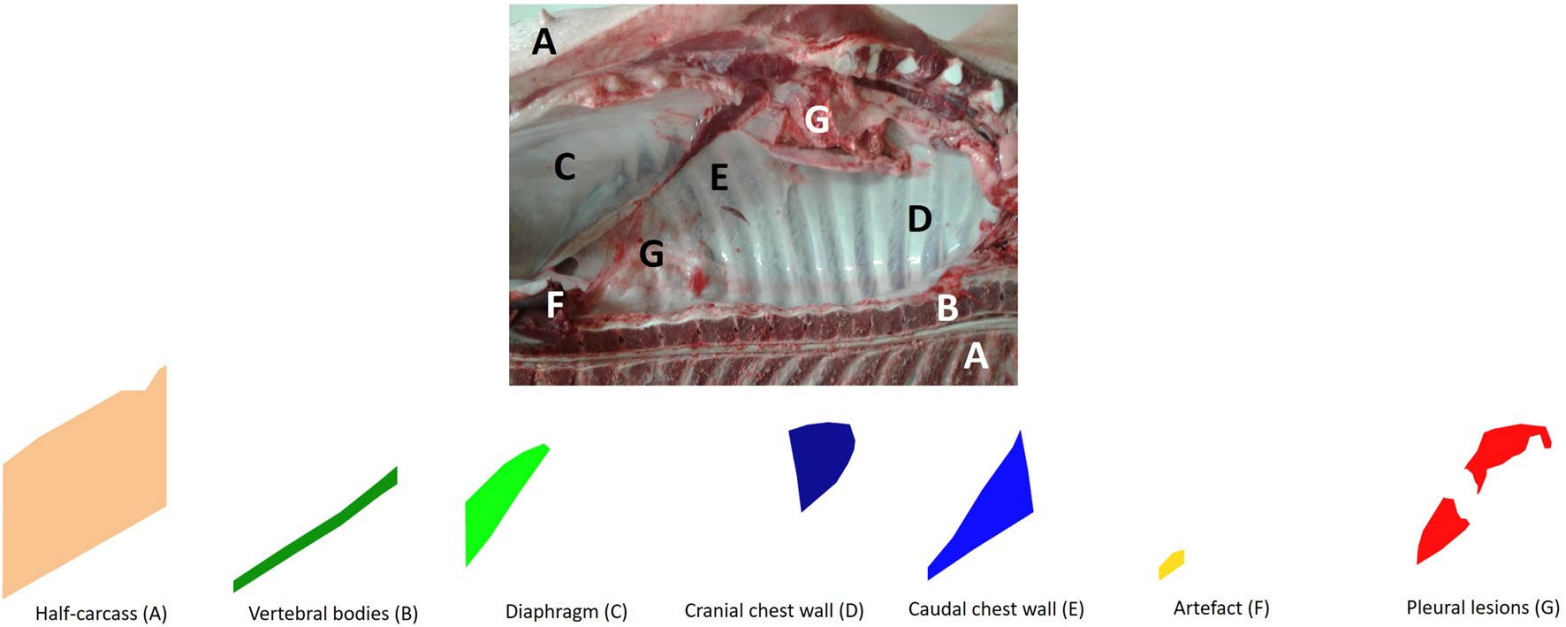
**Layer-wise annotation process: an example.** The DL-based method converted the original image into 7 layers, each one corresponding to an annotated district: half-carcass (**A**), diaphragm (**B**), vertebral bodies (**C**), cranial chest wall (**D**), caudal chest wall (**E**), artefact (**F**), pleurisy (**G**).

During the training stage, annotated images were forwarded to the network and the output segmentations were compared with those provided by the veterinarians (“ground-truth annotation”), in a layer-wise fashion (Figure 4). The network was trained for 100 epochs (each epoch includes all the train dataset examples). Data augmentation, including random translations and rotations, was also performed on each input. During the inference phase, the image is simply forwarded to the network, but no ground-truth comparison is performed.

### Dataset

To train and fairly evaluate the performances of the DL-based method proposed herein, the dataset was split between train and test sets. The former (training set) consisted of 5702 images and was employed during the training stage. The test set consisted of 200 images (i.e. 50 images for each scoring class) and was shown to the network only during the inference stage, when no weight in the network could be altered.

### Scoring the segmentation masks using a rule-based classifier

**Figure 5:**
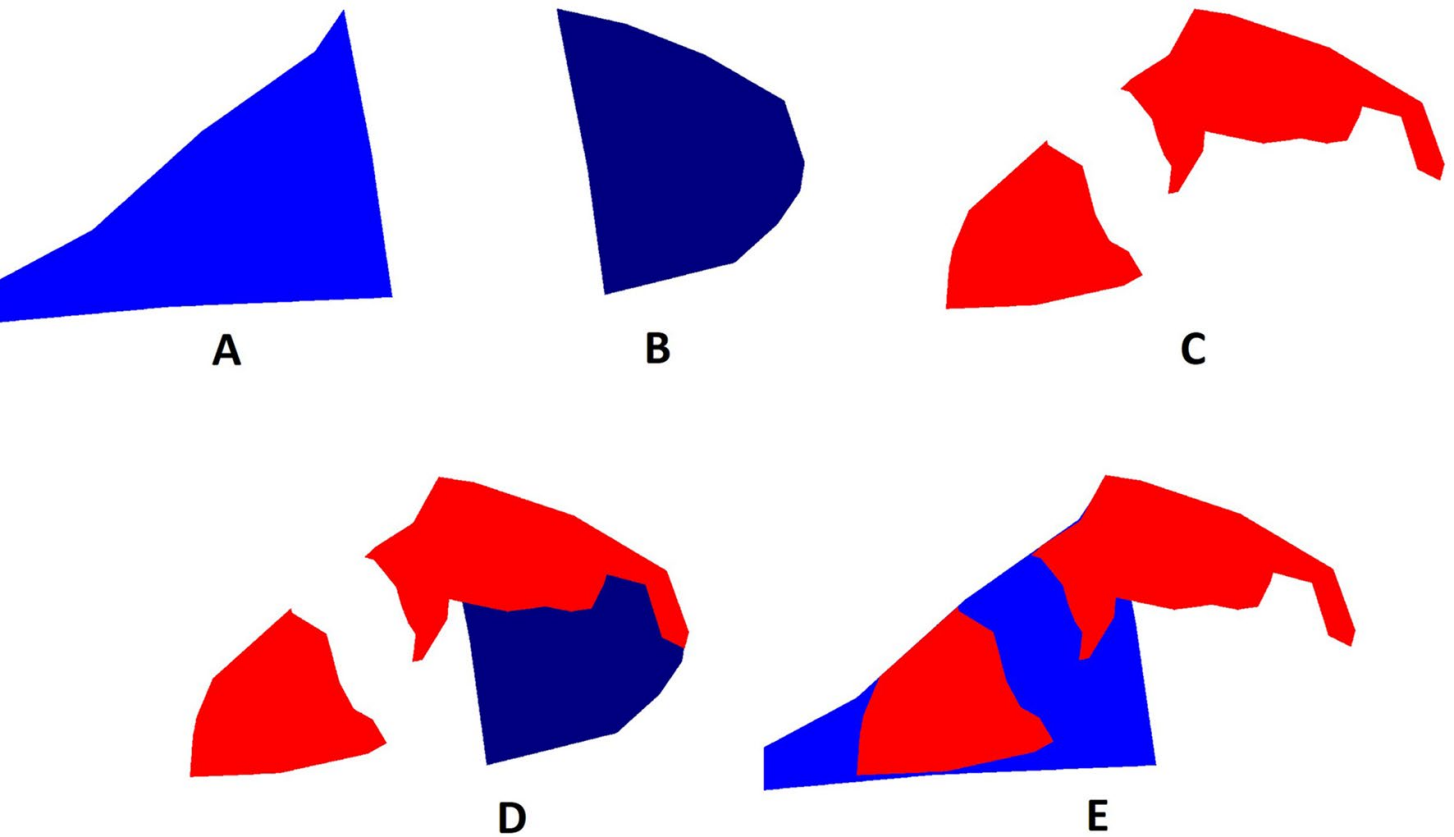
**Scoring through a rule-based classifier: an example.** The rule-based classifier selected 3 layers of the segmented picture, namely caudal chest wall (**A**), cranial chest wall (**B**) and pleural lesions (**C**). Thereafter, the same classifier checked the overlapping among such districts, in order to assign the score: (**D**) the pleural lesion (red color) partially overlaps with the cranial chest wall (dark blue color); (**E**) the pleural lesion (red color) partially overlaps with the caudal chest wall (light blue color). Total score = 3.

A rule-based classifier was employed to convert the segmentation masks, as provided by the trained DL-based method, into scores. Three out of the 7 segmentation layers (i.e. pleural lesions, cranial chest wall and caudal chest wall) were analysed by the rule-based classifier. Lesions were isolated in the correspondent layer using a connected components algorithm. Then, each lesion was compared with the two chest wall layers, in order to check whether there was an overlap (Figure 5). Finally, the rule-based classifier assigned a score, according to the simplified PEPP method, as follows:

- Lesion overlapping with cranial chest wall = 1 point;
- Lesion overlapping with caudal chest wall = 2 points;
- Lesions overlapping with both cranial and caudal chest walls = 3 points.

No further analysis was required in absence of lesions.

### Statistical analysis

The DL-based methods were evaluated in terms of accuracy rates, i.e. calculating the ratio of the number of correct predictions to the total number of input samples. The ability to discriminate between healthy and diseased half carcasses, regardless of the score given, was also calculated as specificity and sensitivity, respectively.

## Results

### Pleurisy scoring provided by the veterinarians on the entire dataset

Pleurisy was detected in 2483 out of 5902 pictures (42.07%), while the remaining 3419 were considered healthy (57.93%). More in detail, according to the chosen simplified PEPP method, 516 half-carcasses scored 1 point (8.74%), 656 half-carcasses scored 2 points (11.11%) and 1311 half-carcasses scored 3 points (22.21%).

### Pleurisy scoring provided by the trained DL-based method

**Figure 6:**
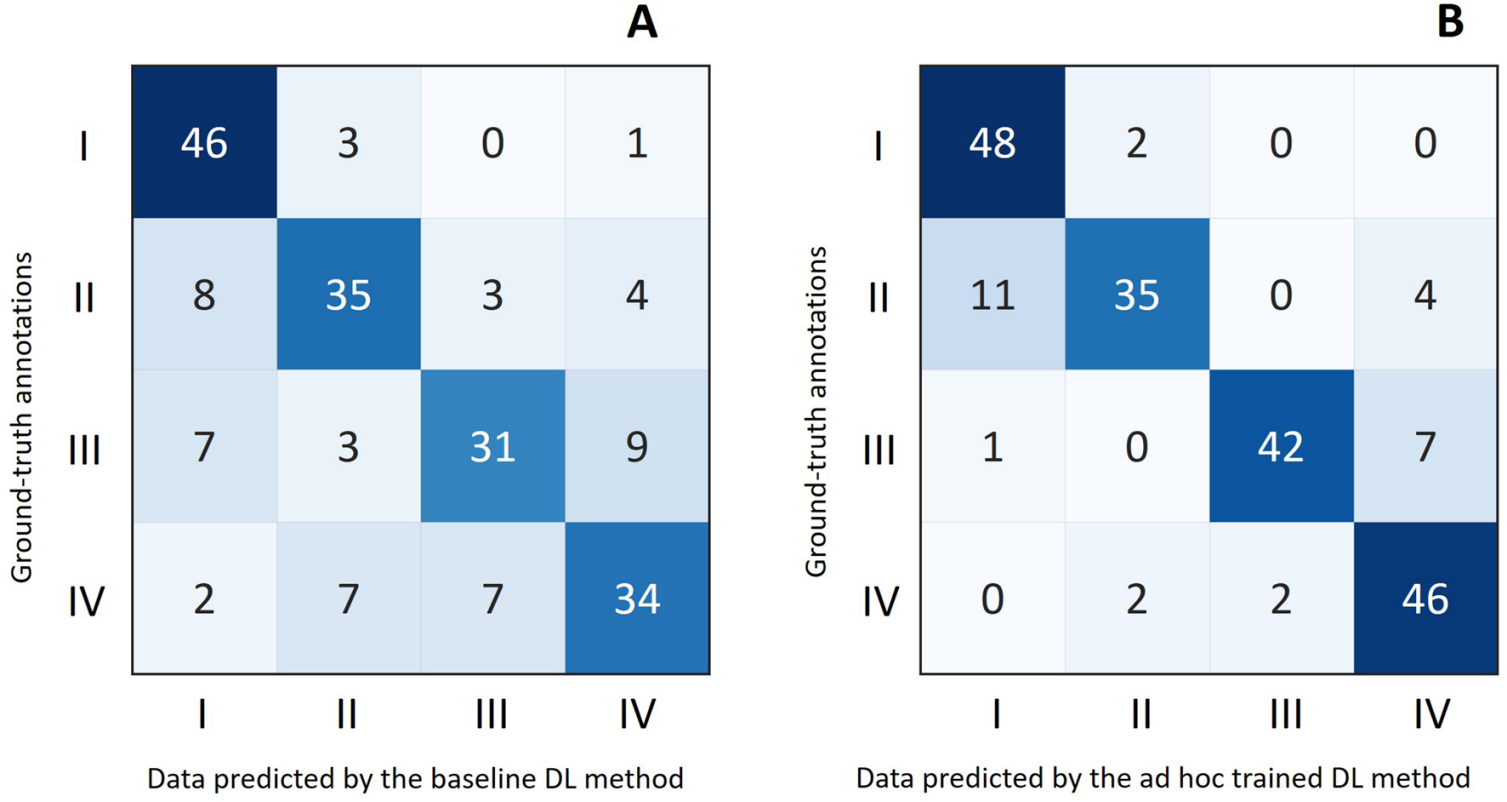
**Test set.** Confusion matrix. Tables compare data provided by the veterinarians (“ground-truth annotation”) with those provided by the baseline DL method (**A**) and by the ad hoc trained DL method (**B**). The diagonal elements represent the correct prediction; the darker the blue color, the more correct the prediction of the DL-based method. The ad hoc trained method (**B**) correctly predicted a very high number of healthy (48/50), class IV (46/50) and class III (42/50) half carcasses. The prediction was less effective for class II lesions (35/50). Overall, the ad hoc trained DL-based method provided better results as compared with those given by the baseline DL method.

As shown in Table 1, the overall accuracy of the ad hoc trained DL-based method on the independent test set (200 images) was 85.5%. This method proved to be very effective at recognizing healthy (accuracy rate = 96%) and diseased half carcasses. More in detail, it was well able to score pleurisy affecting both the cranial and caudal chest wall areas (class IV; accuracy rate = 92%) or affecting only the caudal chest wall (class III; accuracy rate = 84%), lower values being provided for class II lesions (accuracy rate = 70%). The same data is shown in a confusion matrix (Figure 6).

**Table 1:** **Data is shown regarding the accuracy rates of both baseline and ad hoc trained methods.**

Method	Accuracy rates (%)				
	Healthy half carcasses (class I)	Lesions class II	Lesions class III	Lesions class IV	Average value
Baseline DL method	92	70	62	68	73
Ad hoc trained DL-based method	96	70	84	92	85.5

Accuracy values clearly indicate that the segmentation-based approach provides better results, the only exception being represented by class II.

Table 2 shows the specificity and sensitivity values. Obviously, in this case the specificity coincides with the accuracy rate. The overall sensitivity, i.e. the ability to identify diseased half carcasses regardless of the score given, was 92%, and proved to be extremely high for class III and IV lesions (98 and 100%, respectively).

**Table 2:** **Data is shown regarding the specificity and sensitivity of both the baseline and ad hoc trained methods.**

Class	Baseline DL method	Ad hoc trained DL-based method
	Specificity (%)	
I	92	96
	Sensitivity (%)	
II	84	78
III	86	98
IV	96	100
II + III + IV	88.6	92

The value of specificity was very high for both methods. The ad hoc trained method showed very high values of sensitivity for class III and IV, while such value was lower for class II, due to the presence of small lesions which remained overlooked. Also in this case, the ad hoc trained method provided better results, the only exception being represented by class II.

The ad hoc trained DL-based method was further compared with a “baseline method”, the latter consisting of the same CNN structure combined with a four-class classifier. The baseline method employed only input images and output scores, suffering from the lack of segmentation data (i.e. annotated pictures) even when the entire training set was provided. As shown in Tables 1 and 2, the baseline method was less well performing; in particular, the lowest accuracy rates were obtained for classes III and IV lesions (62.0% and 68.0%, respectively), while the lowest sensitivity values were gained for classes II and III (84% and 86%, respectively).

## Discussion

Artificial intelligence-based technologies are very topical, intriguing and virtually able to revolutionize most human activities. It is widely accepted that AI will radically reshape the competitive dynamics of many industries, greatly impacting the economic development of countries, as well as the nature of human work [27]. The application of AI technologies in the field of biomedical sciences is particularly lively and promising. In this respect, it should be remarked that over 18 000 articles are currently available in the US National Library of Medicine National Institutes of Health by typing “artificial intelligence” and “medicine” as keywords, covering most of the disciplines of human medicine, including pathology [28]. On the contrary, very few papers have yet been published regarding the application of AI to veterinary pathology [29–32]. Interestingly, Sanchez-Vazquez et al. [31] applied a machine learning methodology to identify associations among different disease conditions in slaughtered pigs, the scoring carried out by swine veterinarians acting as their data source. Very recently, McKenna et al. [32] applied machine learning to detect pericarditis and hepatis parasitic lesions at post-mortem inspection in pigs.

The SPES grid clearly distinguishes between cranioventral and dorsocaudal pleural lesions, as the latter are worldwide recognized as indicative of previous *A. pleuropneumoniae* infections. A large body of evidence indicates that the *A. pleuropneumoniae* index (APPI), a parameter provided by the SPES grid and which specifically considers dorsocaudal pleurisy, closely relates to the presence and severity of porcine pleuropneumonia in the herd of origin [9, 14]. Although following a different approach, the PEPP method demonstrated to strongly correlate with SPES, thus representing an alternative tool to score pleurisy, also applicable on digital images [15].

To the best of our knowledge, the present study represents the first application of AI technologies to detect and quantify lesions in slaughtered pigs. Overall, our data indicates that the trained CNN is able to discriminate healthy from diseased pleural surfaces. In particular, data provided by the CNN almost fully overlap with those resulting from the application of the gold standard method (i.e. the scoring carried out by swine veterinarians), where healthy and severely affected half-carcasses are concerned. The trained CNN showed lower accuracy values for intermediate scores (classes II and III), reasonably due to the following main factors:

- The lower number of half-carcasses scoring 1 and 2 compared with those scoring 0 and 3 that were used to train the AI system. We consider that this issue could be properly solved by increasing the number of observations, considering that the CNN can “feed itself”, thus progressively improving its performance;
- The presence of small lesions straddling the 5^th^ intercostal space, not easily interpreted even by veterinarians. In this respect, we consider that CNNs could represent an added value, providing more standardized results and thus cutting out the natural inter-operator variability, which is among the most relevant concern of the current scoring methods.

The training of CNNs to identify and score other pathological conditions (EP-like lesions, in primis), on the one hand, and the achievement of trials in large capacity slaughterhouses, on the other, represent the natural pursuance of the present study. Preliminary data, obtained after the training with 3200 pictures, indicate that the ad hoc developed CNN already shows a high accuracy rate (92%) in discriminating between healthy and diseased lungs. However, such values are much lower if we consider the DL-based method’s ability to correctly predict the dimension of pneumonia (accuracy rates ranging between 29.75 and 80.57%) and must be improved for a suitable score of EP-like lesions.

As a result, AI-based technologies could provide a fast and cheap tool to systematically record lesions in slaughtered pigs, thus supplying an enormous amount of useful data to all stakeholders in the pig industry. In particular, such data would represent a useful feedback for the farmers, as well as an effective stimulus to improve herd management, as suggested by the available scientific literature [31]. We consider that the development and application of AI-based technologies will deeply modify the professional life of veterinarians, without affecting their key role to suitably interpret data and to implement the best disease control strategies. Moreover, the massive body of data obtained through AI-based technologies could be used for epidemiological investigations, on a regional, national or international scale, resolutely moving toward evidence-based medicine [11].

In conclusion, our data indicate that CNNs can be effectively trained to diagnose and score lesions in slaughtered pigs. This would allow the systematic collection of data at slaughter, making available an enormous amount of data, useful for better health management of livestock. We consider that we are very close to the umpteenth, epochal revolution in the field of veterinary medicine. Veterinarians should be able to face such challenges, using new technologies, to improve their professional activity.

## Data Availability

The datasets used and/or analysed during the current study are available from the corresponding author on reasonable request.

## Abbreviations

PRCD: porcine respiratory disease complex
*M. hyopneumoniae*: *Mycoplasma hyopneumoniae*
*A. pleuropneumoniae*: *Actinobacillus pleuropneumoniae*
EP: enzootic pneumonia
SPES: slaughterhouse pleurisy evaluation system
PEPP: pleurisy evaluation of the parietal pleura
AI: artificial intelligence
DL: deep learning
CNN: convolutional neural network
APPI: *Actinobacillus pleuropneumoniae* index
